# Characterizing the near-road NO_x_ gradient for exposure assessment purposes

**DOI:** 10.1016/j.epm.2026.01.002

**Published:** 2026-01-19

**Authors:** Jessica L. Levasseur, Qingyu Meng, Kristen M. Rappazzo, Peter Byrley

**Affiliations:** U.S. EPA, Center For Public Health and Environmental Assessment, Research Triangle Park, NC 27711, United States

**Keywords:** Traffic-related air pollution (TRAP), Nitrogen oxides (NOx), Environmental contamination, Distance from roads, Exposure

## Abstract

**Background:**

Nationwide, in 2020, 24 % of the U.S. population lived near high volume roadways, a source of nitrogen oxides (NO_x_) and nitrogen dioxide (NO_2_) emissions. This study synthesizes literature regarding NO_x_ and NO_2_ gradients in the near-road environment to understand community exposures. We synthesize literature reporting measured NO_x_ and NO_2_ concentrations at increasingly large distances from a single roadway and variables impacting those concentrations.

**Methods:**

Using the systematic literature search devised by U.S. EPA to develop Integrated Science Assessments, we identified publications that focused on near-road gradients of NO_2_ and NO_x_. The literature around near-road decay gradients were synthesized and summarized. Concentrations, distance to roadway, and other relevant information were extracted and analyzed using visual and regression methods to better understand the near-road decay gradient.

**Results:**

Concentrations of NO_x_ and NO_2_ decrease with increasing distance from major roadways, following nonlinear decay patterns. Analyzing these data revealed a decreasing power decay trendline. NO_x_ and NO_2_ concentrations typically reduced approximately to background levels by 500 m, with the steepest decline occurring within 50 m from roadways. Concentrations increased with cooler temperatures and higher traffic.

**Conclusion:**

Gradients characterized by this review described how near-road populations are exposed to NO_x_ and the variables that may better describe community exposure. This review provides a quantitative research synthesis of variables most impactful on near-road gradient exposures to NO_x_. Various meteorological and traffic variables were found to impact near-road gradients, though the gradient was driven by distance from the road.

## Graphical Abstract

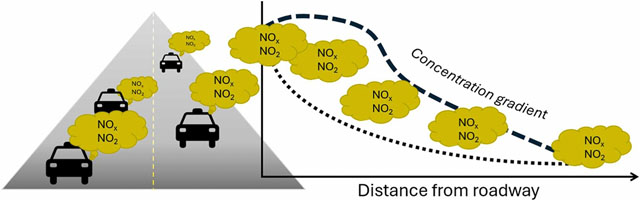

## 1. Introduction

Exposure to ambient oxides of nitrogen is associated with a variety of human health outcomes across the lifespan, including asthma, hospitalization, and mortality [1–4]. Individuals living in urban areas are more likely to be exposed to higher nitrogen dioxide (NO_2_) levels compared to less urbanized areas [5], and differences in exposure levels by social and economic factors have been reported [6–8]. As of the 2020 census, 24 % of the U.S. population lived within 500 m of a roadway with at least 25,000 annual average daily traffic (AADT), over 500,000 people live within 100 m of roads with more than 200,000 AADT, and 21 % of the U.S. population lived within 500 m of a roadway with at least 500 heavy-duty vehicles (HDV) AADT [9].

While nitrogen oxides (NO_x_) can generally refer to the larger group of highly reactive gases made up of oxides of nitrogen, it is dominated by NO_2_ and nitrogen oxide (NO) in near-road settings. In this manuscript, NO_x_ will refer specifically to the sum of NO and NO_2_. NO_x_ concentrations vary spatiotemporally throughout regional/urban areas and microenvironments, are typically associated with traffic emissions, and can be affected by industrial sources, shipping channels, and local environmental conditions [1]. The 2016 Integrated Science Assessment (ISA) for Oxides of Nitrogen, a comprehensive review and assessment published by the U.S. Environmental Protection Agency to support the National Ambient Air Quality Standard for oxides of nitrogen, reported that near-road NO_x_ concentration gradients caused by mobile source emissions played a large role in urban spatial gradients [1]. Higher concentrations of NO_x_ in urban areas have been associated with higher traffic volumes, densities, counts, and loads [10–28]. The relationship between traffic (alternatively defined as traffic volume, traffic type, or traffic counts, depending on the study) and NO_x_ concentration is not linear [1,17], though the exact form of this gradient is uncertain.

Understanding the spatiotemporal gradient moving away from a roadway is essential to understanding NO_x_ exposure(s) experienced by communities who live near roadways. NO_2_ has historically been used as an indicator of traffic-related exposure, especially in urban areas [1,9]. There is good scientific agreement that elevated NO_2_ typically extends up to 500 m from roads with heavy traffic [29]. There is also evidence that influences from the road on NO_2_ concentrations can extend farther, up to several kilometers from the road, but with smaller differences in concentration [29–32]. Functions fitting NO_2_ concentration data as operations of distance from the road have been characterized as logarithmic models, exponential decay models, and shifted power law models [33–35]. Further, the shape of these gradients is affected by meteorology, vehicle count, topography, and other atmospheric compounds including ozone, which reacts with NO to form NO_2_ and is also formed by photolysis of NO_2_ [1,17,18]. With the plethora of factors affecting the gradient that defines near-road exposures, it is critical to characterize these gradients in a more systematic fashion. A generalized gradient for exposure assessors and risk assessors throughout the United States is important for truly understanding human health risks that may be associated with near-road exposures to oxides of nitrogen such as NO_2_ and NO_x_.

The objective of this work is to collate and systematically synthesize recent existing studies that characterize NO_2_ and NO_x_ gradient decay with distance from roadways. Given continuing decreasing national trends in NO_2_ concentrations and changing vehicle fleet mix due to an increase in vehicle electrification technologies, we felt it was important to reevaluate near-road gradients based on the most updated published concentration measurements in literature and compare different measured oxides of nitrogen concentrations at multiple distances from roadways [36,37]. This includes an investigation of the distance at which NO_2_ or NO_x_ reach ambient background levels, sources of oxides of nitrogen and factors that affect this gradient, and a summary of the general spatial gradient moving away from roadways to better estimate human exposure to oxides of nitrogen. This manuscript is presented in two parts: first, a review of the literature to understand which data are available for better understanding the near-road gradient concentrations of NO_2_ and NO_x_ and the factors that may influence these gradients. Second, an aggregation of the data found in the literature review to create a simple near-road concentration gradient. These analyses should be useful to both exposure assessors and risk assessors in their attempt to qualitatively and quantitatively describe exposure and risk associated with NO_2_ and NO_x_ concentrations in a near-road environment throughout the contiguous United States.

## 2. Methods

Methods below describe both the literature search and the quantitative analysis from the literature review results. Each are described separately below, either as “Literature Review” or “Quantitative Near-Road Gradient Assessment.”

### 2.1. Literature review methods: data sources, search strategy, and eligibility criteria

Following approaches described in Volume 2 of the 2024 Integrated Review Plan for the Primary National Ambient Air Quality Standards for Oxides of Nitrogen (NOx IRP) [38], a literature search of the exposure science research regarding oxides of nitrogen published between March 2014 and June 2023 was conducted. The literature search strategy outlined in the NOx IRP was intended to capture all updated scientific literature relating to human exposure to oxides of nitrogen as an update to the 2016 Integrated Science Assessment for Oxides of Nitrogen (2016 Oxides of Nitrogen ISA); however, it also served as way to specifically capture the most recent literature on near-road gradients for oxides of nitrogen. Though the overall search was much wider in scope than our needs, we utilized these comprehensive search results as a starting point for our review.

Briefly, a forward citation search using the citations from the Source to Exposure chapter of the 2016 Oxides of Nitrogen ISA [1] as citation seeds and a keyword search were performed using the PubMed and Web of Science databases; examples of the keywords used in the search can be found in Table S 1. Both a forward-citation and keyword search were performed because they identified documents using different methodologies. The keyword search identified literature based on similar terms. The forward-citation search identified literature based on similar citations and may better capture relevant literature that uses diverse language [39]. These methods have been recommended as part of systematic review methodology [40] and are used as part of the ISA development process to maximize precision and recall of the literature search [41].

Keyword searches consisted of three keyword groupings connected by the “AND” Boolean operator: 1) chemical names and synonyms; 2) air exposure pathway terms; 3) terms relevant for ISA exposure chapter sub-topics. From the search results, authors Q.M., J.L., and P.B. screened at the title/abstract level with SWIFT Active Screener (SWIFT AS, as described by Howard et al. [42]) using a pre-defined scoping statement, i.e., STEM statement, specified in Table 1. Full-text screening was then performed using DistillerSR (DistillerSR. Version 2025.7.2. DistillerSR Inc.; 2023. Accessed May – August 2024. https://www.distillersr.com/) and study quality was also assessed using principles outlined in Section A.5.4 of Volume 2 of the 2024 NOx IRP [38].

From the identified literature, papers that were primarily focused on near-road exposures (defined herein as at least within first 100 m from the road) were further screened for gradient characterization and analysis. Publications that detailed the gradients of NO, NO_x,_ and/or NO_2_ were included in this analysis only if they detailed measuring concentrations at three or more distances from the same road. Aggregated gradients, such as those using national-scale data at various distances from different roads, were not considered. We focused on measured concentrations rather than modeled concentrations, as we expect measurements to yield concentration data under real-word conditions, using these as input parameters to derive empirical concentration gradients.

### 2.2. Literature review methods: data extraction

Data from the peer-reviewed manuscripts were extracted into Microsoft Excel spreadsheets for regression analysis to characterize the near-road gradient concentration decay. Extracted data included geographical study area, date of study, concentration averaging time, gradient distances captured, and factors that were thought to possibly influence concentrations such as time of year, wind direction, wind speed, relative humidity, temperature, and traffic information. PlotDigitizer [43] was used to extract concentration data at various distances from the roadway from figures in Saha et al. [44], but all other data were from tables provided in the manuscripts. NO and NO_2_ data were reported in ppb, and most NO_x_ data were also reported in ppb. Any NO_x_ data reported in μg/m^3^ was converted to ppm assuming ambient conditions (i.e., 1 atm and 25 °C) for statistical analyses using the molecular weight of NO_2_ (46 g/mol) as a conversion factor.

### 2.3. Quantitative near-road gradient assessment: statistical analysis

Gradients were investigated utilizing concentration and distance data. For this analysis, we required studies to have concentration measurements for at least three distances from each roadway. Data from all relevant publications were plotted by distance and multiple regressions were performed to find the best-fit line based on the collected data. Regressions were investigated visually, and an iterative process was used to determine the best fit line for the data, using goodness of fit statistics of R^2^, p-values, and root mean squared error (RMSE). Using R (version 4.4.1), a spline-like regression model was used to characterize the reported NO_2_ and NO_x_ near-road concentration gradients moving away from roadways. Simple linear and exponential decay functions were also fit to the data. A sensitivity analysis was performed on all regression parameters, including spline knot location and number of knots, to investigate goodness of fit using RMSE, p-values (alpha = 0.05), and R^2^ values.

## 3. Results

**Fig. 1.**
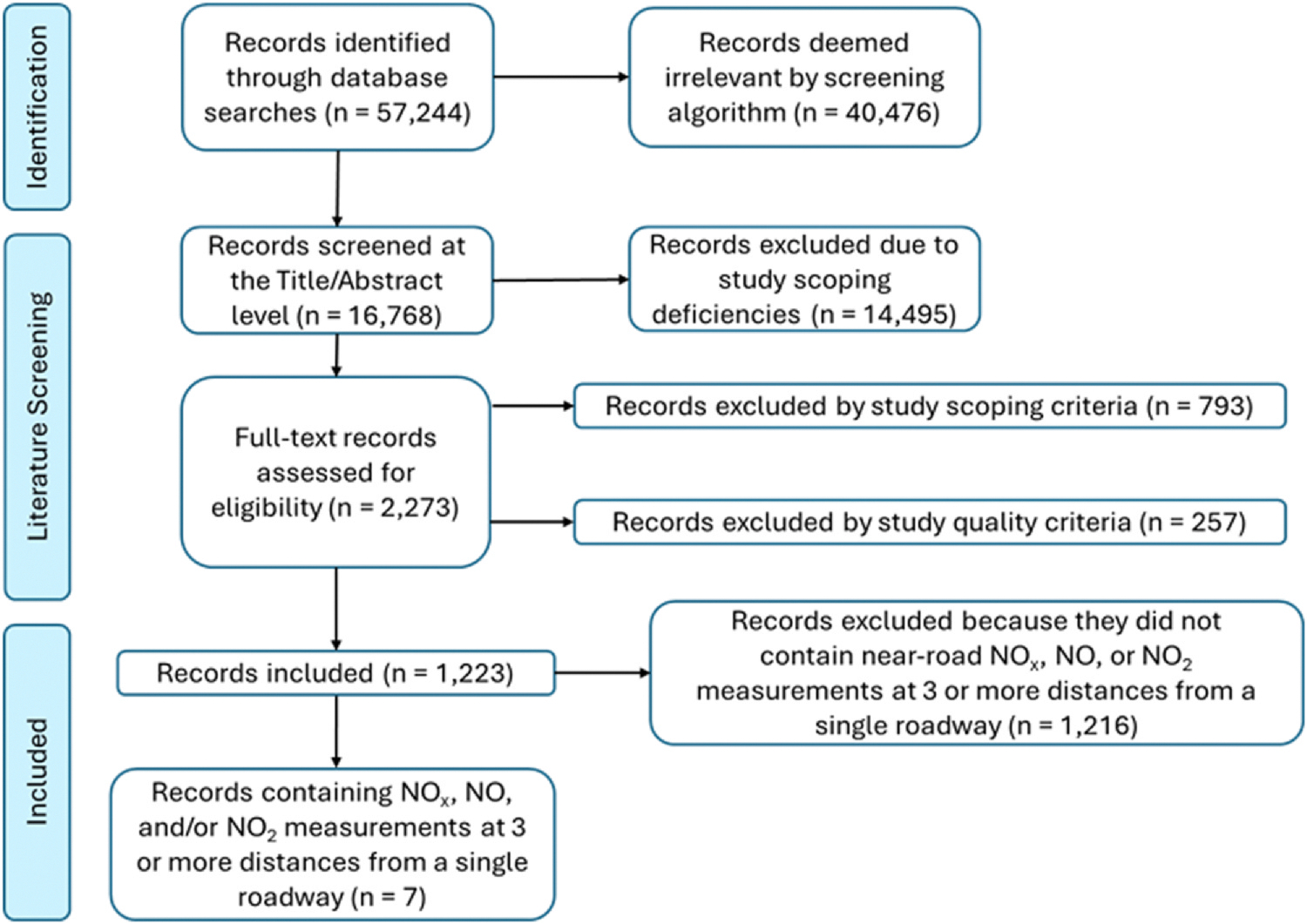
Literature screening workflow.

The literature flow diagram Fig. 1 details the results of the full-text literature screen.

### 3.1. Literature review: included literature

Seven peer-reviewed studies were published between March 2014 and June 2023 detailing measurements of NO_x_ at three or more distances from a single roadway. All studies included were conducted in North America, with most in the United States. Three of the studies were conducted year-round, while four were seasonally limited. Across studies, averaging times for concentrations varied from one minute to one year. The road distances investigated ranged from 10 m to 2.3 km [15,17,18,44–47]. Those studies evaluating the concentration gradient are summarized in Table 2 and discussed below. In Table 2, “near” is defined as the closest monitor to the road, while “far” is defined as the farthest monitor from the roadway.

### 3.2. Literature review results: variables that impact near-road gradients

#### 3.2.1. Traffic

Traffic volume and composition are known variables that impact the concentration of oxides of nitrogen, as NO_2_ concentration has often been used as a surrogate for traffic-related exposure [1]. Herein, “traffic” is defined as either traffic volume, traffic composition, or traffic counts. Higher traffic, especially heavy-duty vehicles, leads to greater emissions and steeper gradients. NO_2_ was observed to have some dependence on AADT [44,45,47], though this dependence was also observed to depend on seasonality [44,47], fleet mix [17,45,47], wind speed and direction [45], and speed of traffic [45]. In a study where data were constrained to only those measurements captured while downwind, traffic volume was most important accounting for 6.8 % of the variance for NO_2_ and 16.5 % for NO_x_ [18]. Generally, as traffic increased, NO_x_ concentrations near the roadway increased. Traffic counts were reported to be a significant factor for NO_2_ concentration decreases from the roadway [15], though traffic volume, rather than counts, was more commonly cited as a significant factor by other publications. Fleet mix, or the traffic composition, was also impactful, as diesel vehicles are associated with higher emissions of oxides of nitrogen compared to vehicles with non-diesel engines [1]. The impact of traffic composition and volume vary temporally as different times of the day are usually accompanied by different wind conditions. For example, during the evening rush hour, 3–8 p.m., when more thermally-driven turbulence and dispersion occurs due to increased traffic, weaker correlations were observed between NO_2_ measured at roadside and at other sites [15]. Though most of the studies summarized herein were investigating emissions on highways [15,17,44,45,47], local land surface arterial roadways were also investigated [15,17,45]. AADT values ranged greatly across and within studies, from 6990 to 353,700 [15,17,44–47]. The percentage of heavy-duty vehicles ranged from 6 % [44] to 10 % [17], while diesel vehicles were reported to range from 1.6 % to 12.0 % per day [45] Table 2.

#### 3.2.2. Wind speed and direction

Both wind speed and wind direction impact near-road gradient concentrations for NO_x_, with concentrations typically higher downwind and under low wind speed conditions where stable atmospheric conditions contribute to less vertical mixing and dispersion and thus steeper gradients. In a Detroit-based study, NO and NO_x_ concentrations decreased by half when downwind by about 130 m from the roadway and to background (upwind) levels by 350 m [45]. In an Atlanta-based study, Liang et al. [15] examined effect modification of variables such as wind speed and wind direction on distance-driven gradients. They scaled the outdoor pollutant concentrations to 200 m for greater physical interpretability. In that study, wind direction was seemingly more impactful than wind speed, as in an Atlanta-based study downwind concentrations dissipated by nearly 9 % for NO_2_ and 17 % for NO per 200 m from the roadway, while in contrast wind speed had a smaller impact on the spatial decay gradient, with increasing wind speed resulting in 2.87 % and 1 % decreases in outdoor NO and NO_2_ concentrations, respectively, per 200 m [15]. Statistically significant concentration decreases of 67 % were observed for NO as wind speed increased from < 3 m/s to > 5 m/s by Baldwin et al. [45], though wind speed was not observed to be significant when investigated in other studies. In Richmond-Bryant et al. [17] concentrations of oxides of nitrogen were measured when monitors were downwind from the major roadway at distances of 10–150 m. The 1-minute NO, NO_2_, and NO_x_ concentrations decreased by 61 %, 20 %, and 61 %, respectively across this distance. These decreases in NO, NO_2_, and NO_x_ were larger than the differences observed between the same distances during upwind conditions (no change, 1 ppb, and 1 ppb, respectively) or those observed under stagnant air conditions (−6 ppb, −2 ppb, and −8 ppb, respectively) [46].

#### 3.2.3. Temperature and temporality

Regarding variations in temperature over time, three of the gradient papers investigated the impact of temperature and/or season on concentration gradients of oxides of nitrogen moving away from roadways [15,18,46]. Two of these studies were conducted in parts of the United States where winter seasonal temperatures are mild [15,18]. Decay rates from the road were influenced by seasonal differences between summer and winter, though concentrations still decreased with increasing distance from the roadway. NO_x_ decayed faster in the summer than in the winter. Average, seasonal hourly NO_X_ concentrations were highest in winter and lowest in spring [47]. Similarly, one study reported that the relative humidity increased the concentration of both NO and NO_2_ by 0.05 % per 200 m from the roadside, while temperature decreased the concentration of NO and NO_2_ by 1–2 % per 200 m away from the roadside, per 10 °F (approximately a 5.6 °C increase) [15]. Diurnal and seasonal variations in traffic patterns were observed, as detailed by Saha et al. [44], where average vehicles per hour reported in Durham, North Carolina ranged from 6900 vehicles per hour in the summer and 5800 vehicles per hour in the winter [44]. Higher concentrations of NO_x_ were observed during lower temperatures across studies [17,44]. Decreases in NO_x_ were observed as temperature increased by Jeong et al. [46] from −15 °C to 10 °C on weekdays, during morning rush hour, in downwind conditions. Using a logit equation to model the gradient in Las Vegas, NV, Richmond-Bryant et al. [18] demonstrated that time of the day contributed to 15.8 % of the variance for NOx, but only 3.3 % for NO_2_. However, time of day contributed more when constrained to only datapoints captured when monitors were downwind: 18.9 % for NO_x_ and 5.8 % for NO_2_. Temperature, however, did not change concentrations considerably for NO_x_ and NO_2_ when considering all data or constraining data to downwind datapoints only (NO_2_: 3.0 %, 2.7 % when downwind; NO_x_ 1.2 %, 2.8 % when downwind) [46].

Differences in roadway gradients were observed between weekend and weekday for both NO and NO_2_, though greater differences were observed for NO (12.46 % decreases on weekday, and 15.01 % on weekends, per 200 m from roadway) [15]. Another study detailed that, when measured at 200 m from the roadside, NO and NO_2_ concentrations varied with time of day with the greatest concentration decrease for NO (17.12 %) during mid-day and for NO_2_ (8.68 %) during mid-day [46]. This is a further example of how temporality can affect concentrations, and as temperature is known to vary throughout the day, how temporality and temperature are linked. Further, Richmond-Bryant et al. [17] reported steeper NO_2_ gradients in the morning and late afternoon compared to overnight in Las Vegas. Jeong et al. [46] reported that downwind NOx concentrations increased by a factor of 2–3 as ambient temperature decreased from 5 °C to 15 °C to −5 °C to −15 °C near Highway 401 in Toronto.

Beyond the temporality of when sampling is collected, how sampling is conducted matters temporally. Four of the seven identified papers utilized shorter sampling times (< 1 h averages), while the other three reported longer sampling times (24 h or annual averages). The data collected using shorter sampling times could capture more variations in concentrations, due to the increased granularity of data measured. This may make detecting a signal or trend more difficult, or alternatively could drive the overall trend to better capture concentration peaks and valleys.

#### 3.2.4. Reported near-road gradient distances

Though near-road spatial gradients are a function of wind direction and speed, traffic, and the presence other chemical species which may interact with NOx in the atmosphere, including O_3_ [17,18], the limited data detailed herein seem to demonstrate distance as the primary driver of concentration gradient changes. The near-road gradient for NO_2_ and NO_x_ observed near Interstate-85 in Las Vegas, NV as part of Richmond-Bryant et al. [17] displayed a negative slope with increasing distance from the road and NO_2_/NO_x_ ratios showed a positive slope as NO was converted to NO_2_ as air moved away from the interstate. Liang et al. [15] investigated the impact on measured NO and NO_2_ concentrations of using monitors at different outdoor distances (10 m, 20 m, 1.4 km, and a 2.3 km urban background site) from the intersection of Interstates-75 and −85 on the Georgia Institute of Technology campus, with AADT of approximately 350,000 vehicles. The authors reported that NO_x_ levels generally decreased farther away from the 10 m site with NO having a stronger gradient than NO_2_, which the authors attribute to interactions of NO with O_3_. Hourly-resolved spatial gradients were also stronger with higher wind speeds and during times with greater traffic counts (10 a.m.−3 p.m.). Liang et al. [15] further confirms that increasing distance away from a road and differences in daily meteorology and traffic count can lead to changes in NO_2_ or NO concentrations.

Multiple studies reported non-linear decay patterns [15,17,18,45,46], with some mathematically modeling both logit [17] and exponential [44,45] fits to their measured concentration decreases curves. Studies that did not fit a formal mathematical model to the data also observed non-linear decay patterns [15,46,47]. Baldwin et al. [42] and Liang et al. [15] observed that NO_2_ concentrations did not exhibit as strong a spatial gradient compared to other primary pollutants, with moderately homogeneous levels from roadside to far-road sites in Atlanta. These gradients were modified temporarily based on factors such as traffic patterns, wind speed, thermally-driven turbulence, and time of day. Factors such as temperature also affected NO_x_ emissions, and cold temperatures can lead to elevated NO_x_ concentrations [46,47]. NO_x_ concentrations decreased by 40 % from 20 to 500 m from the roadside, with average concentrations attenuating slower than other criteria air pollutants such as PM_2.5_ for the first 200 m, and then faster after 200 m [15]. Gradients were modeled for distances from 10–220 m [44], 20–300 m [17], 50–500 m [45] from the major roadways. As compared to studies from Detroit [45] and central North Carolina [44], lower concentration attenuation rates were observed in Houston within the first 200 m [47]. Concentration attenuation was accelerated when monitoring sites were downwind from the highway, and was affected by seasonality, with steeper gradients observed with cooler temperatures. Some studies indicate the steepest concentration declines within the first 20 m of a roadway for certain species, such as NO [15], though distances up to 350 m are typically required to reach background for oxides of nitrogen in general [44,45].

### 3.3. Near-road gradient overall trend analysis

NO_x_ concentrations are generally observed to decrease with increasing distance from a major roadway. However, error in estimation of concentrations between monitors increases with increasing distance between monitors, resulting in the need for modeling techniques to estimate personal exposure of a population [7,17]. Extensions of exposure concentrations by statistical modeling techniques are generally more essential in rural settings, where monitoring locations are more sparsely distributed.

#### 3.3.1. Results: quantitative near-road gradient assessment

The results of the statistical analyses of the reported gradients are detailed below, which attempts to aggregate and fit the reported data to an equation. Due to inconsistent data being collected across studies included in this review, only distance from the road was considered for this gradient analysis as it was the only consistently measured metric across included studies (Table S 3).

**Fig. 2.**
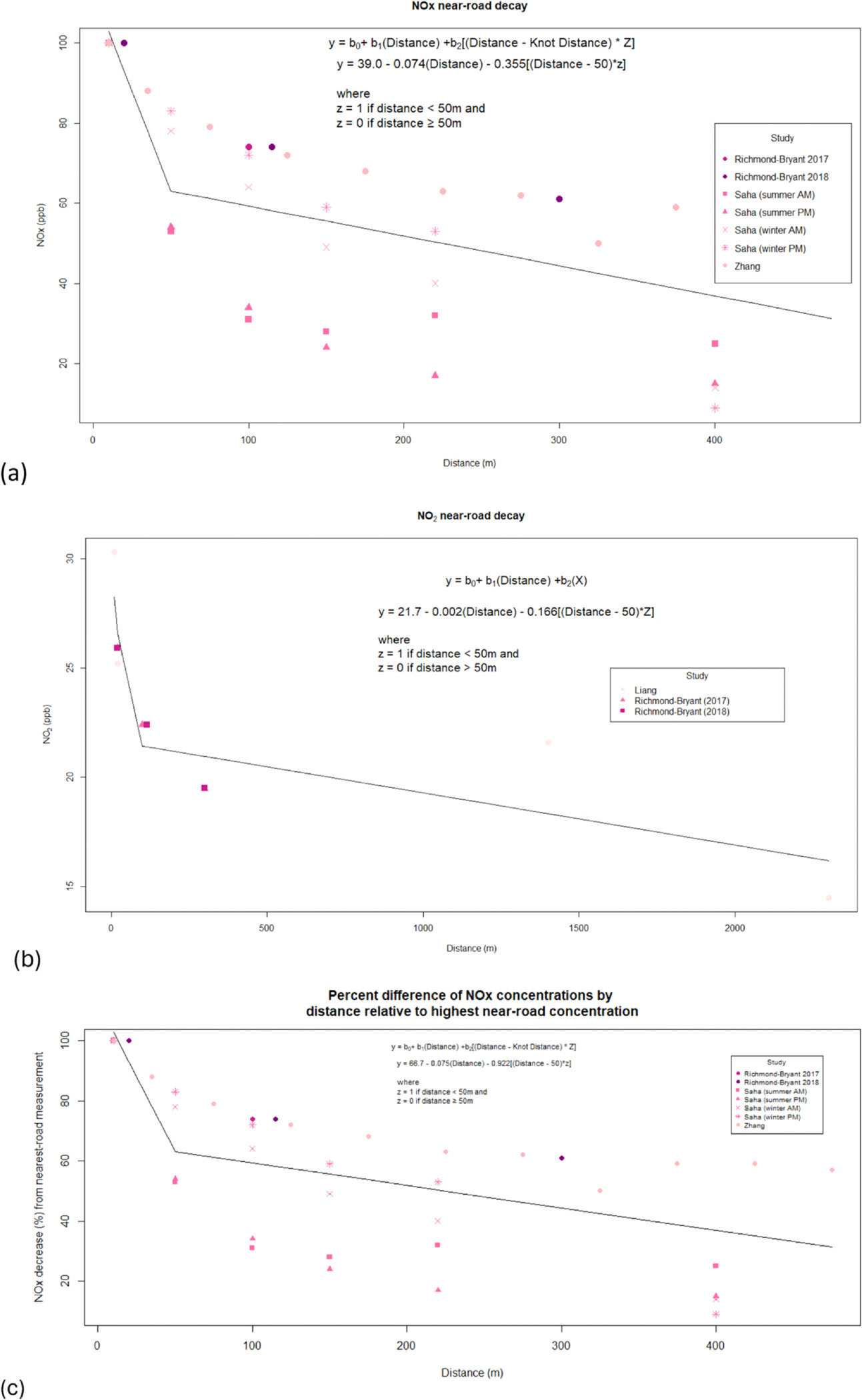
Near-road concentration gradients for NOx (a) and NO_2_ (b) and modeled decay equations. Plot (c) shows near-road concentration gradients for NOx and decay equations, normalized by highest concentration per study.

The concentration gradient determined by this review generally decreases with increasing distance from the roadway. After dropping Jeong et al. [46] from the meta-analysis gradient investigation due to only measuring two distances from each roadway, one manuscript remained that contained NO data. Therefore, there were only enough data to perform a meta-analysis on NO_2_ and NO_x_. As shown in Fig. 2, a sharp decrease is observed within the first approximately 50 m from the roadway, while a more gradual descending gradient continues as far as was measured by the studies detailed herein for both NO_2_ and NOx. A sensitivity analysis investigating the goodness-of-fit for linear, polynomial, log, and spline-based regressions to describe the near-road concentration gradient are detailed in Table 3. This analysis began with a simple linear model as a baseline, graduating to models with increasing complexity such as log and polynomial models to explore best fit. Due to the difference in concentration gradient observed within 50 m from the roadways, a spline-based regression model was also investigated.

As demonstrated by the statistical analysis detailed herein, concentrations in these gradients decayed to background-like levels by approximately 400 m for NO_x_, as demonstrated by concentrations comparable to upwind concentrations from the same roadway or by decaying by at least 75 %. For NO_2_, the decay to background was observed to take about 2300 m by Liang et al. [15], where a decay by 52 % was observed. No other study herein measured NO_2_ beyond 300 m from a roadway.

The distance-response gradients, in this case investigating the concentration of NO_2_ or NO_X_, were systematically analyzed by investigating R^2^, p values, and RMSE for the resulting gradients based on the knot placement(s). A detailed explanation of these can be found in Table S 2. Briefly, models were generally stable regardless of knot choice. However, 50 m from the roadway was chosen as the most appropriate approximate knot point because of both its simplicity and the best agreement between goodness-of-fit values. A generalized equation is demonstrated in Equation 1, where y is the concentration of oxides of nitrogen of interest.

Equation 1. Generalized near-road concentration gradient spline for NO_2_ and NO_X_, where Z is a constant representing if the distance is greater than or equal to the knot (0) or less than the knot (1)

$$y=b_{0}+b_{1}(\mathrm{Distance})+b_{2}[(\mathrm{Distance}\;-\;\mathrm{Knot}\;\mathrm{Distance})\times Z]$$

As demonstrated pictorially in Fig. 2, a single knot was deemed best based on the general fit of the data, the amount of data points, and the overall visual trend. The details regarding the NO_x_ near-road gradient decay are found in Equation 2 below and shown in Fig. 2a. For NO_x_, a 50 m knot resulted in a spline with an R^2^ of 0.257, a p-value of < 0.001, and an RMSE of 20.909. A further investigation of these decaying concentrations normalized to the highest concentration observed can be found in Fig. 2c. As observed in that normalized plot, there is a closer distribution of data points nearer to the roadways, and greater variability as distance from the roadway increases. Our choice of a knot at 50 m is further strengthened by the data distribution observed in this normalized figure, with a more drastic jump in normalized concentrations after 50 m from the roadway.

Equation 2. NO_x_ Concentration equation based on distance from the road, where Z = 1 if distance is < 50 m, and Z = 0 if distance is ≥ 50 m

$${NO}_{x}Concentration\;=39.0-0.074(Distance)-0.355[(Distance\;-50)*Z]$$

For NO_2_ (Fig. 2b), a single knot at 50 m was demonstrated to also be the best approximation of the general near-road gradient. The simplicity of a single knot to describe general oxides of nitrogen near-road gradients was helpful for generalizability and was also useful due to the goodness of fit measures: R^2^ = 0.802, p value = 0.001, RMSE = 0.392. Though a single knot at 20 m was observed to have better goodness of fit values, the two of the three sets of NO_2_ datapoints plotted had a 20 m measurement, perhaps artificially increasing the goodness of fit of a single knot at 20 m. In contrast, a review of literature in the 2016 Oxides of Nitrogen ISA found a larger difference in NO_2_ concentration within the first 10–20 m away from roads than farther distances, suggesting that an inflection point at 20 m may be equally as valid [1]. Additionally, these fits were created with few datapoints available, as demonstrated by the similarity of model fits across the different knot number and placements (Table S 2). However, goodness of fit values observed at 50 m were comparable, as generally these models were stable across knot choices. Equation 3 below details the decay gradient described for NO_2_ concentrations.

Equation 3. NO_2_ Concentration equation based on distance from the road, where Z = 1 if distance is < 50 m, and Z = 0 if distance is ≥ 50 m

$${NO}_{2}Concentration\;=21.7-0.002(Distance)-0.166[(Distance\;-50)*Z]$$

#### 3.3.2. Discussion: quantitative near-road gradient assessment

Near-road concentration gradients generally are impacted by distance from the roadway, traffic volume and composition, and wind speed and direction. These are critical details that should be considered to assess near-road concentration gradients for exposure considerations. Other factors that influence near-road concentration gradient are temporality, such as seasonality or time of day/week, and meteorology, such as wind speed and direction. Those studies with higher AADT values or hourly vehicle counts were associated with more urban areas (Atlanta, GA [15]; Las Vegas, NV [17]; Detroit, MI [45]; Toronto, Canada [46]), while lower values were measured in suburban areas such as Durham, NC [44].

In addition to varying spatially, NO_x_ concentrations also varied temporally, demonstrating concentration trends by season, weekday, and hour. NO_2_ exhibits strong seasonal variability with higher concentrations in summer and lower concentrations in winter. NO_x_ concentrations in urban areas have been demonstrated to be lower on weekends than on weekdays, with this trend weakening due to lower anthropogenic emissions [48,49]. Goldberg et al. [50], using TROPOMI (TROPOspheric Monitoring Instrument) satellite data across the continental U.S., found agreement with earlier studies that normalized mean NO_2_ concentrations were generally lower across cities measured on weekends compared to weekdays [51]. These temporal differences will affect exposure over time, and thus temporality of exposure should be considered during exposure assessments and/or risk assessments to NO_2_ and NO_x_ when considering near-road concentrations. However, across the seven included studies identified by the literature search above, only distance from the road was consistently measured and recorded.

In addition to temporal variations due to temperature, concentration of oxides of nitrogen also varied across the week and within each day based on traffic patterns. As noted earlier, the influence of traffic is further impacted by seasonality, fleet mix, traffic speed, temperature, and wind speed and direction. The influence of these variables is further demonstrated by Kendrick et al. [13] which observed that in the morning hours, a 100-vehicle increase in a 15-minute period was associated with a 1.2–2.5 ppb increase in NO_2_, depending on the season. Traffic varies throughout the day and week, which must be considered in the context of diurnal and annual temperature variations as well. The 2016 Oxides of Nitrogen ISA [1] reported that NO_2_ concentrations near and far away from roads are influenced by season and time of day. As detailed by Liang et al. [15], hourly NO_2_ concentrations were higher 10–15 m from the road than 80–100 m regardless of location, season, or time of day, and that near-road influence was largest during the day in warmer months and smallest at night in winter NO_2_ concentrations were observed to increase during morning rush hour, gradually decrease from late morning to mid-afternoon, and then increase again during evening rush hour and nighttime, but the near-roadway gradient was largest in the afternoon hours [52]. As temperature, wind movement, traffic volume, and fleet composition vary throughout the day, these factors may account for some of the differences observed based on temporality of measurement [46].

Without consistent collection of these factors that may affect the near-road gradient, however, it is impossible to incorporate them into a concentration gradient model effectively. It would be beneficial for studies to collect information on near-road gradient concentrations beyond distance from the road and concentration at that distance. These variables include wind speed, wind direction, traffic information, and information on temporality. Though other factors may also affect this gradient, they were not collected or reported often enough in the literature for a thorough investigation into their effects. With more information, a more detailed gradient equation could be ascertained describing the factors that may affect near-road NO_x_ concentrations.

### 3.4. Overall strengths and limitations

Limited data is available for near-road NO_x_ and NO_2_ exposure concentrations for US populations, particularly for the individual components of NO_x_, which is comprised of mostly NO and NO_2_. Many adults and children, including those who are in lower economic brackets, are often subject to higher near-road concentrations of oxides of nitrogen due to their proximities to roadways [53]. Though this higher exposure has been observed, few recent studies have attempted to measure, with at least three measurements in a straight line from a single roadway, the concentration gradient of oxides of nitrogen from roadway emissions. Through our systematic review detailed above, seven recent publications detailed concentrations from a single roadway at multiple distances in the same direction. Table 2 shows that six out of seven studies had six or fewer measurements along the gradient. This sparse data is understandable given the cost associated with spatially variable measurements from a single roadway. Further, with the limited number of studies available for our analysis, average concentrations across various averaging times were combined, while ideally concentrations from similar averaging times would be more directly comparable. With recent advances in low-cost sensors for saturation sampling of NO_x_ [54], there is considerable room for future publications to investigate this gradient more granularly, as understanding this gradient allows communities to better protect their near-roadway communities more effectively. There is ample future opportunity to investigate these near-road concentration gradient trends with more granularity as low-cost sensors become more viable and common. Investigations such as these will be increasingly important moving forward, as the gradient may likely also change because of a changing vehicle fleet with the increasing number of hybrid and zero-emission electric vehicles on U.S. roadways. A future, finer investigation will be needed to reassess near-road community exposure potential [55].

A limitation of our gradient investigation is that because of the sparse data available, results from different locations around North America had to be combined. Though each data set represents one near-road scenario, each scenario, as detailed by Table 2 and Table S 3, had different meteorology, traffic, and geographical conditions which affected the shape of the gradient in that individual dataset. More information would allow for investigations of joint effects of these factors on the near-road concentration gradient, which we were unable to thoroughly investigate herein. Examples of the factors that study authors investigated across included manuscripts can be found in Table S 3. Due to the inconsistencies across studies regarding gradient-affecting factors captured by each analysis, we were unable to incorporate many of these variables into our quantitative statistical analysis of the near-road concentration gradient. If future studies capture factors known to affect concentration gradients in the near road environment, such as wind speed, wind direction, temperature, humidity, and traffic-related information, these details can be used in future quantitative characterizations of the near-road concentration gradient. We recommend that future study designs measuring concentrations at multiple distances near roads try to capture as much information as reasonably allowed related to meteorology, traffic conditions, and geographic conditions at spatial and temporal scales appropriate to support evaluation of near-road measurements. Though we were still able to determine a best overall fit, more granular data from each test site would be ideal, especially for NO, to better characterize the concentration-distance relationship. Further, the knot locations in the spline models were likely affected by the monitor locations. The Y-intercept (b_0_) value was likely affected by the differences in overall concentrations between studies. The position of the spline knot would likely not be affected by overall concentration as there seems to be a more dramatic drop within the first 50 m, across studies, compared to concentrations measured at a farther distance. With increased granularity in concentrations at more distances moving away from major roadways, the regression will be better informed and less driven to fit by the common measurement distances.

A strength of this manuscript is that it is the first review to systematically investigate the oxides of nitrogen literature to generalize a concentration gradient for near-road exposures to NO_2_ and NO_x_. The overall literature will benefit from the data that we have pooled herein, representing different dispersion conditions across meteorological effects and geography, and how these gradients can be understood given the near-road monitoring network results. Subsequently published literature may further add to our investigation of the general concentration gradient experienced in near-road environments. The sensitivity analysis detailed herein adds to the confidence we have in this description as a starting point for future investigations of near-road gradient exposures to oxides of nitrogen.

## 4. Conclusion

Understanding near-road gradient of traffic-based pollutants helps practicing risk assessors and exposure assessors better characterize potential NO_2_ and NO_x_ exposures to nearby communities. Without understanding of this gradient, and at what point concentrations are decreased, higher exposures cannot be mitigated. This gradient is affected by many meteorological factors that vary throughout the country, such as temperature and wind, and affected by traffic. As all three of these factors vary throughout the day, week, and year, understanding temporality differences for these near-road gradients is another crucial variable necessary in future near-road concentration gradient investigations. Our analysis herein will be improved with the addition of more investigations into concentration gradients of traffic-related air pollutants like oxides of nitrogen, which should be analyzed at smaller distance increments to fully understand the gradient and external factors that may affect this gradient.

## Supplementary Material

Supplement

## Figures and Tables

**Table 1:** STEM statement to define the criteria and framework for identifying relevant oxides of nitrogen exposure studies [38].

Statement	Description

**Source (S)**	Emissions from ambient (e.g., traffic) or non-ambient (e.g., cookstove emission) sources of oxides of nitrogen.
**Transport and Transformation (T)**	Atmospheric and environmental processes of oxides of nitrogen, including the transport of air pollutants at various scales (i.e., national/global, regional, urban, neighborhood, middle, micro scales, and microenvironments), including near-source (e.g., near traffic) transport and transformation, and advances in chemical transformations and deposition from the atmosphere (e.g., photochemical reactions) and microenvironments (e.g., indoor chemistry).
**Exposure/extent (E)**	Exposure levels of oxides of nitrogen characterized by various surrogates (e.g., ambient concentrations, microenvironmental concentrations, personal exposure and exposure determinants (i.e., factors which may lead to differential exposures, such as proximity to sources, activity patterns, and socioeconomic status), including characterizing concentrations and spatiotemporal temporal trends of various exposure surrogates and examining populations experiencing elevated exposures or the exposure patterns (e.g., exposure level, duration, and frequency) experienced by populations identified in health studies as being at increased risk of effects.
**Measurement and Modeling (M)**	Measurement methods (e.g., federal reference and equivalent methods, passive samplers, sensors, and remote sensing) and modeling techniques (e.g., land use regression and dispersion models) characterizing ambient air, indoor/microenvironmental air, and personal exposures, including the evaluation of measurement principles and modeling assumptions, examination of potential bias and uncertainties, and comparison of different techniques.

**Table 2:** Summary of near-road gradient investigations for oxides of nitrogen.

Study ID, Location	Traffic Count (vehicles/time)	AT	Time of Year	Oxides of Nitrogen Measured	C_near_ Average (ppb#)	C_far_ Average (ppb#)	Distance From Road (m)

Baldwin et al. [45], **Detroit, Michigan, USA**	Range: 25,963–167,800 vehicles/d	5 min	Winter (December 2012)	NO	C_diff_ Average: 14.7*		50 m
				NOx	C_diff_ Average: 17.1*		150 m
				NO_2_	C_diff_ Average: 5.8*		500 m
Jeong et al. [46], **Toronto, Canada**	Average: 11,000 vehicles/hr	1 min	Winter (February 2017)	NO	23	9	10 m
				NOx	47	29	150 m
				NO_2_	24	20	
Liang et al. [15], **Atlanta, Georgia, USA**	Average vehicles/d: Highway: 353,700 Arterial roadway: 6990	24 hr and 1 hr	Winter (September 2014 – January 2015)	NO	20.9	15.7	10 m
				NOx	NA	NA	20 m
				NO_2_	30.3	14.5	1.4 km 2.3 km
Richmond-Bryant et al. [17], **Las Vegas, Nevada, USA**	Average: 206,000 vehicles/d	1 hr	Year-round (December 2008 – January 2010)	NO	NA	NA	20 m
				NOx	51.0	31.3	100 m^a^
				NO_2_	25.9	19.5	100 m^b^ 300 m
Richmond-Bryant et al. [18], **Las Vegas, Nevada, USA**	Average: 206,000 vehicles/d	1 hr	Year-round (December 2008 – January 2010)	NO	NA	NA	25 m
				NOx	51.0	31.3	115 m^a^
				NO_2_	25.9	19.5	100 m^b^ 300 m
Saha et al. [44], **Durham, North Carolina, USA**	Average: 6350 vehicles/hr†	1 hr	Summer 2015, Winter 2016	NO	NA	NA	10 m^c^
				NOx	44‡ (50 m)^◊^	24‡ (220 m)^◊^	50 m
							100 m
				NO_2_	NA	NA	150 m 220 m 400 m^b^
Zhang et al. [47], **Houston, Texas, USA**	Not Reported	annual	Year-round (January – December, 2011)	NO	NA	NA	0–20 m
				NOx	25.73 μg/m^3^	16.56 μg/m^3^	20–50 m
							50–100 m
				NO_2_	NA	NA	100–150 m 150–200 m 200–250 m 250–300 m 300–350 m 350–400 m 450–500 m

AT = averaging time; NO = nitric oxide; NOx = NO_2_ + NO; NO_2_ = nitrogen dioxide.

† Calculated average based on seasonality averages in vehicles/hour.

# All concentrations reported in ppb unless otherwise noted.

* Only C_diff_ (C_far_ – C_near_) reported.

‡ Fig. 2 extracted using PlotDigitizer [43], averaged across morning, afternoon, and season.

a downwind.

b upwind.

c fixed trailer.

◊ Saha et al. [44] used 50 m as the beginning of the near-road gradient, and 400 m as an upwind, background monitor.

**Table 3:** Fit equations and sensitivity analysis results for NO_2_ and NO_x_ (where y = concentration, x = distance, and z = 1 if x < 50 m and z = 0 if x ≥ 50 m).

	NO_2_				NO_x_			
Fit	*Equation*	*R* ^*2*^	*P-value*	*RMSE*	*Equation*	*R* ^*2*^	*P-value*	*RMSE*

Regressions								
Linear	y = 24.6−0.004x	0.447	0.021	2.928	y = 46.4−0.098x	0.275	0.00003	20.853
polynomial (2)	y = 22.7−9.412x + 0.902x^2^	0.374	0.081	2.914	y = 51.7−0.188x + 0.0002x^2^	0.281	0.00010	20.557
polynomial (3)	y = 22.7−9.412x + 0.902(x^2^) − 6.832(x^3^)	0.671	0.021	1.956	y = 54.9−0.302(x) + 0.001(x^2^) − 1.1e10^−6^(x^3^)	0.274	0.00030	20.450
Log	y = 31.6−1.902(log(x))	0.627	0.004	2.403	y = 83.9−11.67 (log(x))	0.301	0.00001	20.482
1 knot (50 m) Spline	y = 21.7−0.002x − 0.166[(x-50)*Z]	0.802	0.001	0.392	y = 39.0−0.074x − 0.355[(x-50)*Z]	0.257	0.00133	20.909

Though all regressions were observed to be significant at p < 0.05, the RMSE were generally consistent across the different fit equations. R^2^ values were generally highest when fitting the data to a 1-knot spline or log decay curve.

## Data Availability

Not Applicable.

## Appendix A. Supporting information

Supplementary data associated with this article can be found in the online version at doi:10.1016/j.epm.2026.01.002.
