# Supplementary material for: Characterizing the near-road NOx gradient for exposure assessment purposes
Source: Environ Pollut Manag. Author manuscript; Available in PMC 2026 May 7. (PMC13137458; doi:10.1016/j.epm.2026.01.002)
Supplement: Supplement [file NIHMS2150710-supplement-Supplement.docx]

**Supplemental Information for: Characterizing the near-road gradient for NO_x_ for exposure assessment.**

Jessica L. Levasseur^a^, Qingyu Meng^a^, Kristen M. Rappazzo^a^, Peter. Byrley^a*^

^a^U.S. EPA Center For Public Health and the Environment, Research Triangle Park, NC, 27711

*Corresponding Author

**Table S 1. Examples of the keywords used in the 2016 ISA literature search**

| **Topic** | **Search String** |
| --- | --- |
| Example chemical names and synonyms | HNO(2), HNO(3), HNO2, HNO3 , N(2)O , N2O , nitric acid , nitric oxide , nitrogen oxides , nitrogen dioxide , nitrogen monoxide , nitrogen oxide , nitrogen oxides , nitrogen dioxide , nitrous acid , NO(2), NO(3), NO(x), NO(y), NO(z), NO2 , NO3 , NOx , Noy , Noz , organic nitrate , organic nitrates , oxides of nitrogen , peroxyacetyl nitrate , peroxyacetyl nitrates , peroxyacyl nitrate , peroxyacyl nitrates , peroxynitrite , RONO(2), RONO2, nitrogen oxide, nitrogen oxides, oxide of nitrogen, oxides of nitrogen, nitrogen dioxide, nitrogen dioxides, nitrogen peroxide, nitrogen peroxides, nitric oxide, nitric oxides, nitrogen monoxide, nitrogen monoxides, nitrous acid, HONO |
| Example air exposure pathway terms; | air pollution, ambient, monitor, monitoring, monitoring station, network, exposure estimate, exposure metric, exposure model, personal exposure, individual exposure, model, estimated, measure, simulation, indoor, exposure measurement error, exposure error, true exposure, true value, spatial variability, spatial variation, temporal variation, temporal variability, instrument imprecision, inter, intra, individual, personal, exposure, population air, ambient, exposure |
| Example terms relevant for ISA exposure chapter sub-topics | aerosol , PM2.5, PM10, OC, organic carbon, BC, black carbon, EC, elemental carbon, soot, black smoke, PAH, polycyclic aromatic-hydrocarbon, O3, photochemical, Ozone, Hg , mercury, SO2, sulfur, nickel, transition metals, organic aerosol, organic compound, volatile organic compounds, CO, carbon monoxide, exhaust, diesel, traffic, traffic-related air pollution, noise, odor, multiple pollutant, multipollutant, gaseous pollutant, emission, reformulated gasoline, in-vehicle, on road, in-traffic, indoor, outdoor, ambient, urban, street canyon, personal exposure, traffic congestion, vehicles, source, road user pricing, distance, ambient, outdoor, outside, fixed site, central, individual, personal, exposure, population, indoor, inside, room, residential, building, in-vehicle, on road, in-traffic, street canyon, ambient, outdoor, outside, fixed site, residential, building, activity level, activity pattern, time-activity, activity indoor, activity outdoor, people's activities, temporal activity, temporal pattern, spent time, activity data, National Human Activity Pattern Survey, exertion level, human activity database, Consolidated Human Activity Database, indoor concentration, indoor exposure, infiltration , indoor outdoor concentration, gas range, indoor level, apartment, gas combustion, gas cooker, gas cooking, gas appliance, indoor air pollution, indoor air quality, kitchen, oil furnace, coal stove, wood burning stove, kerosene heater, smoking, candle burning, electric heating, space heater, indoor combustion, indoor deposition, gas-phase indoor chemistry |

**Table S 2. Spline near-road concentration gradient sensitivity analysis for NO_2_ and NO_X_ data**

|  | **NO_2_** | | | | **NO_X_** | | | | |
| --- | --- | --- | --- | --- | --- | --- | --- | --- | --- |
| Fit | *Knot locations* | *R^2^* | *P value* | *RMSE* | *Knot locations* | *R^2^* | *P value* | *RMSE* |  |
| spline - 1 knot | 20 | 0.814 | 6.19E-04 | 1.342 | 20 | 0.273 | 6.19E-04 | 20.259 |  |
| spline - 1 knot | 50 | 0.689 | 3.80E-02 | 1.734 | 50 | 0.270 | 6.80E-04 | 20.302 |  |
| spline - 1 knot | 75 | 0.627 | 5.82E-02 | 1.900 | 75 | 0.266 | 7.73E-04 | 20.360 |  |
| spline - 1 knot | 100 | 0.6096 | 0.06481 | 1.94 | 175 | 0.2619 | 0.000866 | 20.41294 |  |
| spline - 2 knot | 20, 100 | 0.829 | 2.34E-02 | 1.151 | 50, 175 | 0.2561 | 0.001747 | 20.27863 |  |
| spline - 2 knot | 50, 100 | 0.829 | 2.34E-02 | 1.151 | 50, 300 | 0.255 | 1.81E-03 | 20.296 |  |
| spline - 2 knot | 20, 300 | 0.8181 | 0.02631 | 1.19 | 20, 175 | 0.2583 | 0.001641 | 20.24826 |  |
| spline - 2 knot | 50, 300 | 0.7969 | 0.03249 | 1.25 | 20, 300 | 0.2581 | 0.001648 | 20.25022 |  |
| spline - 2 knot | 75, 300 | 0.7616 | 0.04402 | 1.36 | 75, 175 | 0.2508 | 0.002023 | 20.35039 |  |
| spline - 2 knot | -- | -- | -- | -- | 75, 300 | 0.251 | 0.002011 | 20.34746 |  |
| spline - 3 knot | 20, 50, 100 | 0.829 | 2.34E-02 | 1.151 | 50, 175, 300 | 0.252 | 3.00E-03 | 20.111 |  |
| spline - 3 knot | 50, 100, 300 | 0.852 | 4.52E-02 | 0.927 | 50, 200, 300 | 0.251 | 0.003125 | 20.133 |  |
| spline - 3 knot | 20, 75, 300 | 0.852 | 4.52E-02 | 0.927 | 50, 100, 300 | 0.2549 | 0.002806 | 20.07743 |  |
| spline - 4 knot | 20, 50, 100, 300 | 0.852 | 4.52E-02 | 0.927 | 50, 100, 200, 300 | 0.239 | 6.06E-03 | 20.070 |  |
| spline - 4 knot | 20, 75, 300, 1000 | 0.852 | 4.52E-02 | 0.927 | 20, 75, 200, 300 | 0.2347 | 0.006705 | 20.126 |  |
| spline - 4 knot | 20, 50, 300, 1000 | 0.852 | 4.52E-02 | 0.927 | 20, 50, 175, 300 | 0.2364 | 0.006431 | 20.10271 |  |
| spline - 4 knot | 50, 100, 300, 1000 | 0.852 | 4.52E-02 | 0.927 | 20, 50, 200, 300 | 0.2353 | 0.006604 | 20.117 |  |
| spline - 5 knot | 20, 50, 100, 300, 1000 | 0.852 | 4.52E-02 | 0.927 | 20, 75, 175, 300, 430 | 0.220 | 1.25E-02 | 20.089 |  |

**Table S 3. Factors investigated for their influence on near-road gradient concentrations, per chemical. Factors measured for their impact on the chemical concentration gradient are marked with "x". Greyed cells signify that a particular chemical was not measured within the listed study.**

|  |  | *Factors measured; NO concentration* | | | | | | | |  | *Factors measured; NO_2_ concentration* | | | | | | | |  | *Factors measured; NO_X_ concentration* | | | | | | | |
| --- | --- | --- | --- | --- | --- | --- | --- | --- | --- | --- | --- | --- | --- | --- | --- | --- | --- | --- | --- | --- | --- | --- | --- | --- | --- | --- | --- |
| **Study** | **Time period** | **Wind Direction** | **Wind Speed** | **Relatively Humidity** | **Dew Point** | **Temperature** | **Distance** | **Number of lanes** | **Traffic Counts** | **Time period** | **Wind Direction** | **Wind Speed** | **Relatively Humidity** | **Dew Point** | **Temperature** | **Distance** | **Number of lanes** | **Traffic Counts** | **Time period** | **Wind Direction** | **Wind Speed** | **Relatively Humidity** | **Dew Point** | **Temperature** | **Distance** | **Number of lanes** | **Traffic Counts** |
| [Liang, et al. [1]](#_ENREF_15) | x | x | x | x |  | x | x | x |  | x | x | x | x |  | x | x | x | x |  |  |  |  |  |  |  |  |  |
| [Baldwin, et al. [2]](#_ENREF_41) |  |  | x |  |  |  | x | x | x | x |  | x |  |  |  | x | x | x |  |  | x |  |  |  | x | x | x |
| [Jeong, et al. [3]](#_ENREF_42) |  | x | x |  |  | x | x | x |  |  | x | x |  |  | x | x | x |  |  |  |  |  |  | x | x | x |  |
| [Richmond-Bryant, et al. [4]](#_ENREF_17) |  |  |  |  |  |  |  |  |  | x | x | x |  |  |  | x |  |  |  |  |  |  |  |  |  |  |  |
| [Richmond-Bryant, et al. [5]](#_ENREF_18) |  |  |  |  |  |  |  |  |  | x | x | x |  |  | x | x |  | x | x | x | x |  |  | x | x |  | x |
| [Saha, et al. [6]](#_ENREF_40) |  |  |  |  |  |  |  |  |  |  |  |  |  |  |  |  |  |  | x |  |  |  |  |  | x | x |  |
| [Zhang, et al. [7]](#_ENREF_43) |  |  |  |  |  |  |  |  |  |  |  |  |  |  |  |  |  |  | x |  |  |  |  |  | x | x | x |

**References**

1. Liang, D.; Golan, R.; Moutinho, J.L.; Chang, H.H.; Greenwald, R.; Sarnat, S.E.; Russell, A.G.; Sarnat, J.A. Errors associated with the use of roadside monitoring in the estimation of acute traffic pollutant-related health effects. *Environ Res* **2018**, *165*, 210-219, doi:10.1016/j.envres.2018.04.013.
2. Baldwin, N.; Gilani, O.; Raja, S.; Batterman, S.; Ganguly, R.; Hopke, P.; Berrocal, V.; Robins, T.; Hoogterp, S. Factors affecting pollutant concentrations in the near-road environment. *Atmospheric Environment* **2015**, *115*, 223-235, doi:10.1016/j.atmosenv.2015.05.024.
3. Jeong, C.H.; Hilker, N.; Wang, J.M.; Debosz, J.; Healy, R.M.; Sofowote, U.; Munoz, T.; Herod, D.; Evans, G.J. Characterization of winter air pollutant gradients near a major highway. *Sci Total Environ* **2022**, *849*, 157818, doi:10.1016/j.scitotenv.2022.157818.
4. Richmond-Bryant, J.; Owen, R.C.; Graham, S.; Snyder, M.; McDow, S.; Oakes, M.; Kimbrough, S. Estimation of on-road NO(2) concentrations, NO(2)/NO(X) ratios, and related roadway gradients from near-road monitoring data. *Air Qual Atmos Health* **2017**, *10*, 611-625, doi:10.1007/s11869-016-0455-7.
5. Richmond-Bryant, J.; Snyder, M.G.; Owen, R.C.; Kimbrough, S. Factors associated with NO(2) and NO(X) concentration gradients near a highway. *Atmos Environ (1994)* **2018**, *174*, 214-226, doi:10.1016/j.atmosenv.2017.11.026.
6. Saha, P.K.; Khlystov, A.; Snyder, M.G.; Grieshop, A.P. Characterization of air pollutant concentrations, fleet emission factors, and dispersion near a North Carolina interstate freeway across two seasons. *Atmospheric Environment* **2018**, *177*, 143-153, doi:10.1016/j.atmosenv.2018.01.019.
7. Zhang, X.; Craft, E.; Zhang, K. Characterizing spatial variability of air pollution from vehicle traffic around the Houston Ship Channel area. *Atmospheric Environment* **2017**, *161*, 167-175, doi:10.1016/j.atmosenv.2017.04.032.
